# Supplementary material for: Fabrication of Electrospun Cellulose Acetate/Nanoclay Composites for Pollutant Removal
Source: Polymers (Basel). 2022 Nov 22;14(23):5070. doi: 10.3390/polym14235070 (PMC9738163; doi:10.3390/polym14235070)
Supplement: Supplementary file 1 [file polymers-14-05070-s001.zip › polymers-2062585-SM.pdf]

polymers

Supplementary data

Fabrication of electrospun cellulose acetate/nanoclays composites for pollutants removal

Petya Tsekova and Olya Stoilova*

Laboratory of Bioactive Polymers, Institute of Polymers, Bulgarian Academy of Sciences, Akad. G. Bonchev St, bl. 103A, 1113 Sofia, Bulgaria; stoilova@polymer.bas.bg
* Correspondence: stoilova@polymer.bas.bg

Received: 13 November 2022 ; Accepted: 21 November 2022; Published: 22 November 2022

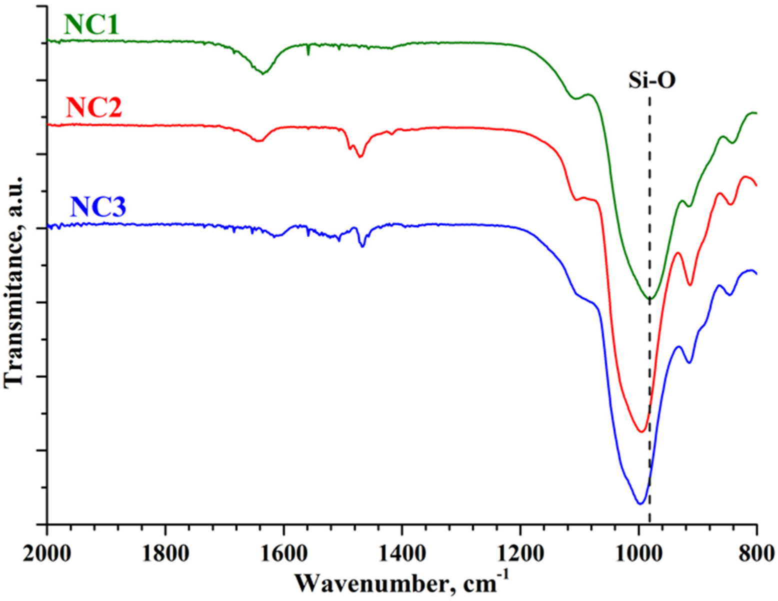

Figure S1. ATR-FTIR spectra of pristine NC1, NC2 and NC3 nanoclays.

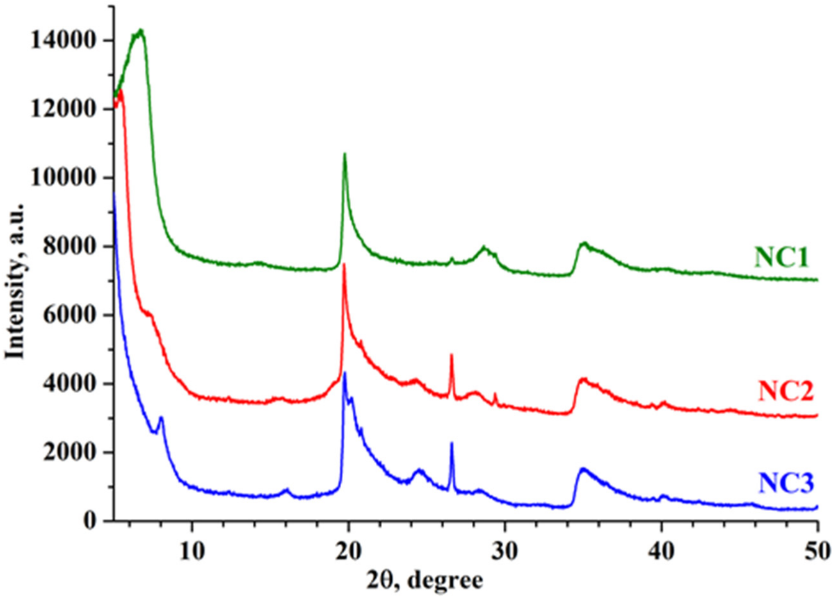

Figure S2. XRD patterns of pristine NC1, NC2 and NC3 nanoclays.
